# An urgent call to raise the bar in oncology

**DOI:** 10.1038/s41416-021-01495-7

**Published:** 2021-08-16

**Authors:** John-John B. Schnog, Michael J. Samson, Rijk O. B. Gans, Ashley J. Duits

**Affiliations:** 1 Department of Hematology-Medical Oncology, Curaçao Medical Center, Willemstad, Curaçao; 2 Curaçao Biomedical and Health Research Institute, Willemstad, Curaçao; 3 Department of Radiation Oncology, Curaçao Medical Center, Willemstad, Curaçao; 4 Department of Internal Medicine, University of Groningen, University Medical Center Groningen, Groningen, The Netherlands; 5 Institute for Medical Education, University Medical Center Groningen, Groningen, The Netherlands; 6 Red Cross Blood Bank Foundation, Willemstad, Curaçao

**Keywords:** Education, Drug development, Drug development, Industry, Research management

## Abstract

Important breakthroughs in medical treatments have improved outcomes for patients suffering from several types of cancer. However, many oncological treatments approved by regulatory agencies are of low value and do not contribute significantly to cancer mortality reduction, but lead to unrealistic patient expectations and push even affluent societies to unsustainable health care costs. Several factors that contribute to approvals of low-value oncology treatments are addressed, including issues with clinical trials, bias in reporting, regulatory agency shortcomings and drug pricing. With the COVID-19 pandemic enforcing the elimination of low-value interventions in all fields of medicine, efforts should urgently be made by all involved in cancer care to select only high-value and sustainable interventions. Transformation of medical education, improvement in clinical trial design, quality, conduct and reporting, strict adherence to scientific norms by regulatory agencies and use of value-based scales can all contribute to raising the bar for oncology drug approvals and influence drug pricing and availability.

## Background

Important breakthroughs have contributed to an increased life expectancy for patients suffering from several cancers, such as immunotherapy for melanoma [1], second-generation androgen receptor antagonists for prostate cancer [2] and new drugs for myeloma [3]. However, reduction in cancer mortality has been mostly due to prevention, early detection and improved cancer care organisation [4, 5]. In contrast, approval of anticancer drugs by regulatory agencies gives the impression that new drugs are drivers of cancer care improvement. In fact, they often offer limited benefits to patients while the costs of many new cancer treatments are skyrocketing [6, 7]. In the Netherlands, the nationwide prospective cancer registry revealed a median increment in the survival of patients with advanced solid cancers of little more than 1 month over the past 10 years [8]. With value in health care defined as the outcome of an intervention relative to its related costs [9], many new oncological treatments are thus of limited value [10].

The increasing array of new treatments in oncology can lead to unrealistic patient expectations and, if not duly managed, pushes society to unsustainable health care costs. With the current COVID-19 pandemic-driven increased necessity to make value-based choices in all medical fields, we should critically reassess the value of available cancer care and allow only high-value interventions based on properly designed and conducted trials to reach our practice. In this narrative review, we focus on different factors that likely contribute to the use of low-value oncological treatments. Strategies for preventing the advent of low-value oncological care are presented.

## Critical interpretation of clinical trials

### Relevant outcomes

The evidence upon which any treatment recommendation is based should ideally come from adequately designed, executed and reproduced phase 3 randomised controlled trials (RCTs). Any treatment should result in clear benefits; improve the chances of a cure or to live longer (increase overall survival [OS]) and better lives (improve quality of life [QOL]) [11]. What absolute OS increase is considered relevant is likely to differ between cultures, age groups and countries. As a frame of reference, to be considered for inclusion on the World Health Organization Model List of Essential Medicines, a cancer drug should increase OS by at least 4–6 months as compared to the standard of care [12, 13]. In contrast, in many modern RCTs leading to drug approvals, median OS improvement is mostly <3 months (Table 1) [14–19]. An example was the approval of erlotinib in the treatment of advanced pancreatic cancer that increased OS by 2 weeks when combined with gemcitabine, as compared to gemcitabine alone [20].

**Table 1:** Overview of major published studies addressing contemporary clinical oncology trials and regulatory drug approvals.

**Overall survival: clinical trials leading to regulatory drug approvals with a limited OS benefit**		
**YOP**	**Timespan study**	**Results**
2014	2002–2014	Solid cancer drug approvals by FDA: 71 approvals with median OS of 2.1 months [16]
2016	2003–2013	62 new drugs approved by both FDA and EMA: 23 (43%) with OS improvement ≥3 months, 6 (11%) <3 months, 8 (15%) unknowns, with no evidence of OS increment in remaining approvals. Mean OS gain of all approvals 3.43 (SD 0.63) months relative to treatment available in 2003 [17]
2017	2009–2013	EMA approved 48 cancer drugs for 68 indications: at the time of market approval, median OS benefit was 2.7 months (range 1.0–5.8) in 24/68 (35%) of indications [14]
2020	2000–2016	First time FDA approval of novel cancer drugs for any type of cancer; 92 novel drugs for 100 indications based on 127 trials: median OS 2.40 months (IQR 1.25–3.89) [15]
2021	2011–2020	pCODR-approved drugs: 78/104 submissions received positive recommendation; median OS gain in approved drugs 3.7 (IQR 2.7–6.5) months as opposed to median OS increase of 1.9 (IQR 1.4–4.5) months in rejected submissions [18]
2021	2010–2020	298 RCTs of systemic treatment in breast, colorectal and NSCLC published in high-impact journals: 86 (29%) had OS as the primary endpoint; in trials with a positive outcome, median OS increase 3.5 (IQR 2.5–6.6) months [19]
**Medical society value-based scales: drugs tested in oncology RCTs are assessed as meaningful in less than half of cases in most studies**		
**YOP**	**Timespan study**	**Results**
2014	2002–2014	Solid cancer drug approvals by FDA: 30/71 (42%) would have met ASCO-CRC threshold for a clinically meaningful benefit to patients (2.5 months and ≥25–30% OS increase), 9/71 (13%) uncertain benefit [16]
2017	2011–2015	All RCTs on breast, pancreas, lung and colorectal cancer: 277 RCTs; 138 (50%) favour experimental group with 43/138 (31%) meeting ESMO-MCB benefit threshold (score 4 or 5 in a palliative setting, score A or B in curative setting) [31]
2017	2011–2015	All RCTs on breast, pancreas, lung and colorectal cancer with significant difference favouring experimental group: 277 RCTs; median ASCO-VF score 25 (range 2–77; score ≥45 considered substantial benefit) and 38% met the ESMO-MCB benefit threshold [30]
2017	2009–2013	EMA approved 48 cancer drugs for 68 indications: 23 associated with OS benefit; 11/23 (48%) were scored as meaningful with the ESMO-MCB scale [14]
2017	2000–2015	All new biologics and molecular entities that were FDA approved; 37/51 (72%) new drugs evaluated: 13/37 (35%) drugs showed meaningful benefit by ESMO-MCB with median ASCO-VF 37 (IQR 20–52) [32]
2017	2006–2016	FDA-approved 63 drugs for 118 indications; 46 (43.8%) met the ESMO-MCB threshold of clinical benefit [33]
2017	2011–2016	EMA approved 38 solid cancer drugs based on 70 studies; 11 and 21% met approval thresholds of adapted and original ESMO-MCB, respectively [34]
2020	2009–2017/19^a^	New drugs for solid cancers approved by both FDA and EMA; 47 drugs for solid cancers; ESMO-MCB 13/47 (28%) met the benefit threshold and 15/36 (42%) met substantial benefit threshold of the ASCO-VF (not applicable to 11 indications) [38].
2020	2012–2017	106 trials led to FDA approvals of 52 drugs for 96 indications: thresholds of clinical benefit were met in 43% of ASCO-VF, 34% of ESMO-MCB, 73% of ASCO-CRC (OS >2.5 months and PFS > 3 months) and 69% of NCCN Evidence Blocks (score 4 and 5 and combined 16 or higher for efficacy, safety, quality and consistency of evidence, affordability) [35]
2021	2011–2020	pCODR-approved drugs: 78/104 submissions received positive recommendation; 61% of accepted submissions considered of benefit based on ESMO-MCBS as to 19.2% in rejected submissions [18]
2021	2006–2019	FDA approved 55 oncology drug indications scorable with ASCO-VF with subsequent publications relevant for reassessing ASCO-VF scoring: at FDA approval 40.0% substantial benefit (score ≥45), 49.1% low (score ≤40) and 10.9% intermediate. At 3 years post approval based on 9 follow-up publications, despite changes in individual scores, 40.0% remained substantial, 50.9% low (score ≤40) and 9.1% intermediate [37]
2021	2006–2017	214 FDA and 170 EMA approvals with 40 and 58% of indications including QOL assessment in trials; using ASCO-VF and ESMO-MCB scales, QOL bonus criteria were detected in 13 and 17% of FDA and 21 and 24% of EMA approvals [36]
**Quality of life: studies show PROs are often not studied and that adherence to CONSORT-PRO guidelines in oncology drug research is not optimal**		
**YOP**	**Timespan study**	**Results**
2015	2007–2011	325 phase 3 RCTs reviewed only 48% of trials reporting PROs. PRO reporting with mean PRO RQS 5 on an 11-point scale [22]
2015	2004–2013	RCTs including PRO endpoint identified in 557 RCTs; <50% reported at least 4/6 CONSORT-PRO items [25]
2016	2003–2013	62 new drugs approved by FDA and EMA: 17 (32%) demonstrated improvement in QOL based on empirical evidence [17]
2017	2009–2013	EMA approved 48 cancer drugs for 68 indications: 9/68 (13.2%) associated with an increase in QOL [14]
2019	2014–2017	Of 160 published RCTs based on NIHR registered protocols with PROs included in endpoints, 61 (38.1%) did not include PROs in any publication. The remaining trials scored a mean of 3 (SD 3) of 14 CONSORT-PRO checklist items in published PRO findings [26]
2019	2004–2019	649 RCTs with PRO reporting, 72 (11.1%) of trials analysed included patients ≥70 years; only 24 (33.3%) had high-quality PRO reporting according to ISQOLR-PRO standards [23]
2020	2014–2019	71 RCTs reporting on PROs in haematological malignancies were identified with the quality of reporting in RCTs employing CONSORT-PRO extension being higher than trials not citing this extension [24]
2020	2011–2018	FDA approved 42 immunotherapy indications with PROs published in 21/44 (47.7%) of the trials. The mean score of a 24-point PRO Endpoint Analysis Score was 12.71 (range 5–27, SD 3.71) [27]
**Surrogate endpoints: oncology trials use of surrogate endpoints**.		
**YOP**	**Timespan study**	**Results**
2011	1992–2010	FDA approved 35 oncology drugs for 47 indications via accelerated approval pathway: all studies based on surrogate endpoints. Conversion to regular approval in 26/47 (55.3%) indications; 16/26 still based on a surrogate endpoint [44]
2014	2005–2012	FDA approved 188 novel agents for 206 indications based on 448 trials; 55/448 are cancer trials of which 83.6% measured a surrogate endpoint [40]
2015	2009–2013	51 FDA approved oncology for 63 indications; 70% of approvals based on a surrogate endpoint [41]
2015	2008–2012	FDA approved 36/54 drugs based on a surrogate endpoint [47]
2017	2009–2013	EMA approved 48 cancer drugs for 68 indications: surrogate endpoint in 53 of 72 RCT’s (73%) [14]
2018	1992–2017	FDA granted AA for 64 drugs for 93 indications; all based on surrogate endpoints [42]
2019	2014–2016	EMA approved 32 new drugs based on 54 trials; 39/41 were published RCT’s with 29/39 (74%) evaluating surrogate endpoints as a primary outcome measure [46]
2019	2006–2018	FDA approved 59 drugs for 85 indications based on RR [39]
2019	1992–2017	AA was granted for 93 cancer drug indications based on surrogate endpoints; confirmatory trials in 19 (20%) measured the same surrogate endpoint, 20 (21%) a different surrogate endpoint, 19 (20%) reported OS improvement with the remaining trials not reported at the time of publication [43]
2021	2011–2020	pCODR approved drugs: 78/104 submissions received positive recommendation; 67.9% surrogate endpoints in approved indications, as opposed to 76.9% in rejected submissions [18]
2021	2016–2020	49 drug approvals by FDA based on 52 trials for haematological malignancies: 84% report surrogate endpoints [45]
2021	2010–2020	298 RCTs of systemic treatment in breast, colorectal and NSCLC published in high impact journals compared to RCTs from 1995–2004 and 2005–2009: PFS as an endpoint: 0 (1995–2004), 18 (2005–2009) and 42% (2010–2020) [19]
**Non-randomised and single-arm studies: drug approvals are increasingly based on single RCTs or non-randomised single-arm studies**.		
**YOP**	**Timespan study**	**Results**
2011	1992–2010	FDA approved 35 oncology drugs for 47 indications via accelerated approval pathway: 28/47 (59.6%) were based on non-RCTs. Conversions to regular approval occurred in 26/47 (55.3%) indications; 24/26 (92%) based on RCTs [44]
2014	2005–2012	FDA approved 188 novel agents for 206 indications based on 448 trials; 55/448 are cancer trials; 52.7% not randomised [40]
2016	1999–2014	76 pharmaceuticals (44/795 EMA, 60/774 FDA) approved without RCT; 34 haematological, 15 oncological indications [56]
2017	2009–2013	EMA approved 48 cancer drugs for 68 indications: 8 (12%) single-arm study [14]
2018	1992–2017	FDA granted AA for 64 drugs for 93 indications; 67 of 93 (72%) indications were based on single-arm trials [42]
2019	2006–2018	FDA approved 59 drugs for 85 indications based on RR: only 9% were RCTs [39]
2020	2000–2016	First time FDA approval of novel cancer drugs for any type of cancer; 92 novel drugs for 100 indications based on 127 trials: 95 (74.8%) nonrandomized [15]
2020	2011–2018	FDA approved 42 immunotherapy approvals; 21/44 (477%) were single-arm trials [27]
2020	2014–2019	187 trials led to 176 approvals by FDA for 75 anticancer drugs; 64 (34%) were single-arm trials [57]
2021	2016–2020	49 drug approvals by FDA based on 52 trials for haematological malignancies: 40% non-phase 3 trials [45]
2021	2011–2020	pCODR approved drugs: 78/104 submissions received positive recommendation; 92.3% phase 3 RCT in approved indications, as opposed to 57.7% in rejected submissions [18]

*YOP* year of publication, *OS* overall survival, *FDA* Food and Drug Administration, *EMA* European Medicines Agency, *SD* standard deviation, *IQR* interquartile range, *pCODR* pan-Canadian Oncology Drug Review, *RCT* randomised controlled trial, *NSCLC* non-small cell lung cancer, *ASCO-CRC* American Society of Clinical Oncology Cancer Research Committee, *ESMO-MCB* European Society of Medical Oncology-Magnitude of Clinical Benefit scale, *ASCO-VF* American Society of Clinical Oncology Value Framework, *NCCN* National Comprehensive Cancer Network, *PRO* patient-reported outcome, *CONSORT* consolidated standards of reporting trials, *RQS* reporting quality score, *NIHR* National Institute for Health Research, *ISQOLR* International Society of Life Research, *RR* response rate, *PFS* progression-free survival, *AA* accelerated approval.

PubMed was searched for all reviews and systematic reviews investigating oncology drugs, approvals, value scales, PRO assessment with publication dates 2000 until the present including search terms such as ‘drugs, approvals, cancer, value, PRO’, and per the study, the ‘similar articles’ link was also assessed. Studies regarding a specific disease indication were not included. Studies were selected according to relevance to the topic of this review.

^a^2009–2017 for FDA and 2009–2019 for EMA approvals.

Improving QOL (assessed in trials as patient-reported outcomes [PROs]) should be the main goal of palliative treatment. Since many trials evaluate surrogate primary endpoints (see below), PRO assessments should be thoroughly and correctly conducted to determine whether the studied intervention delivers meaningful benefit [11]. The Consolidated Standards of Reporting Trials (CONSORT) were extended with specific guidelines to increase the consistency of PRO research and reporting [21]. Several studies have shown that PROs are often not studied and, when addressed, adherence to PRO reporting guidelines is often poor (Table 1) [14, 17, 22–27].

Clinical benefit scales based on factors such as OS, QOL, toxicity and symptom control have been developed to translate outcome measures to value [28, 29]. Several studies have assessed whether oncology drug approvals meet such society-defined thresholds of clinical benefit. Most studies show <50% meeting these standards (Table 1) [14, 16, 18, 30–38].

### Surrogate endpoints

Surrogate endpoints, such as progression-free survival (PFS) or response rate (RR), are increasingly used as primary endpoints in phase 3 oncology RCTs (and in non-randomised studies) (Table 1) [14, 18, 19, 39–47]. Their use may contribute to effective treatments reaching patients faster (e.g. recent immunotherapy and targeted therapy approvals for advanced lung cancer [48]). However, a recent retrospective analysis showed a limited median time gain of ~11 months when PFS is studied as opposed to OS [49]. Surrogate endpoints are mostly poorly predictive of outcome parameters such as OS [50] or QOL [51]. The use of surrogate endpoints can therefore result in patient exposure to treatments without proper evidence of an effect on a clinically relevant outcome. Such was the case with bevacizumab in breast cancer, receiving approval after one RCT revealed a positive effect on PFS [52]. After repeat RCTs failed to confirm the magnitude of the initial PFS benefit, showed no improvement in OS but demonstrated increased bevacizumab-related toxicity [53, 54], the approval was revoked. Notably, this treatment option is currently still included in the National Comprehensive Cancer Network (NCCN) breast cancer guideline [55]. Introducing new treatments based on surrogate endpoints can lead to incorrect ‘new standards of care’, to which future potential treatments are being compared in costly clinical trials.

### Approvals based on non-phase 3 trials

Even though a phase 3 RCT is considered the gold standard for evidence generation, studies spanning more than a decade have demonstrated that a considerable percentage of oncology drug approvals are based on non-phase 3 RCTs [14, 15, 18, 27, 39, 40, 42, 44, 45, 56, 57] (Table 1). Especially, studies leading to expedited approvals often lack active comparators (see ‘regulatory agencies’ below) [58]. For more than half of FDA oncology drug approvals between 2014 and 2019 that were based on single-arm studies, approved therapies already existed to which these should have been compared [59]. Even though subsequent confirmatory phase 3 RCTs are considered obligatory, they are not always performed [42, 43, 47]. When confirmatory trials do address OS as the primary outcome, these often do not show benefit [47]. A recent example was the approval of olaratumab for treatment of advanced sarcoma in combination with doxorubicin based on a 14-month survival increase in a single open-label phase 1b and phase 2 trial [60]. Resources were wasted on a treatment, of which the results of the ensuing phase 3 RCT failed to show OS improvement [61].

### Single RCTs

A new treatment should ideally be approved after demonstrating benefit in a repeated clinical trial. In general, a second positive RCT is considered to confirm the effectiveness of the tested intervention. This is of importance as even with detection of a statistically significant difference between treatment arms in an RCT, there is the possibility of the finding being false positive [62]. Single RCTs are, however, increasingly considered sufficient to lead to drug approvals. A recent study reported that, of 120 FDA approvals (2014–2019), 117 were based on a single RCT [57].

An initially detected effect of an intervention in an RCT is often smaller when the trial is repeated or reanalysed, and large treatment effects in small RCTs are usually not reproducible [63–65]. A recent study showed that post-approval updates of RCTs in breast, lung and prostate cancer show a decrease in the initially detected and reported effect upon which the approval was based [66]. The ‘fragility index’, which is the minimum number of changes from non-events to events causing an RCT to lose statistical significance, could be calculated in 17 of 36 oncology RCTs [67]. An outcome change of a mere two events would have 53% of these 17 RCTs lose statistical significance, casting doubt on their robustness. The chance of a properly conducted RCT being false negative is very small (based on the a priori probability of an intervention being effective and the generally accepted threshold of false negativity in RCTs of 20% (power of 80%)). Taking this all together, drug approvals after a single RCT may be based on false positive, exaggerated and/or ‘fragile’ findings.

### Non-representative for everyday practice

In clinical trials, the selected population and the context of care delivery are commonly not representative of the general patient population or everyday practice. Fit and relatively young patients are included and intensively monitored during treatment [11]. Even though carrying a higher cancer burden, minorities [68] and older patients [23] are often underrepresented, limiting the broad representability of trial findings. The benefit detected in a clinical trial is usually less and toxicity greater in everyday practice, especially in patients who do not fulfil the original inclusion/exclusion criteria of the respective clinical trial. For example, in ‘real-life data’ in both colorectal cancer [69] and prostate cancer [70], it was observed that treatment outcome was worse than expected in patients not fulfilling the original trial inclusion criteria. A recent study compared trial outcomes of 22 FDA-approved oncology drugs for 29 indications to real-world data obtained in older Medicare beneficiaries [71]. Survival was shorter in Medicare beneficiaries in 28 of 29 indications (median difference −6.3 months, range −28.7 to 2.7 months).

### Other important issues in critical evaluation of clinical trials

Many other factors of importance should also be critically assessed when interpreting clinical trials and are beyond the scope of this review. These include the inappropriate use of subgroup analyses [72, 73], the occurrence of inappropriate crossovers [57], the occurrence of informative censoring [74] and the use of inadequate comparator arms [57, 75].

## Bias in data publication

Selective or biased reporting in oncological RCTs occurs even in high-impact journals [76, 77]. Spin bias, i.e., misrepresentation of trial findings with the goal of luring readers into believing that the claimed effect is greater than the available data shown, has been reported in almost 60% of RCTs in breast cancer [78] and in 10% of abstracts pertaining to lung cancer trials [79]. Spin bias in abstracts has been shown to influence clinicians’ trial interpretation [80]. It is also directly translated into lay press reporting [81], leading to unfounded expectations of patients, policymakers and physicians [80]. Publication bias, i.e., non-publication or delayed publication of negative studies, needs to be considered whenever we are addressing a potential new treatment (indication), especially since results of non-published studies could be of relevance [82]. Even though the global cancer burden is highest in low- and middle-income countries (LMICs), publication bias by high-impact journals against LMIC oncology RCTs occurs, even as these are more likely to identify high-value therapies [83]. In systematic reviews of cancer trials, publication bias assessments, such as the use of funnel plots, remain underused, which may therefore exaggerate the effects of the reviewed intervention [84]. Harm detected in clinical trials is often underreported [85].

## Financial conflicts of interest

Identifying new strategies against disease drives academia and pharma. Aside from academic acknowledgement, funding and promotion of clinical researchers, new treatments, or drug repurposing against both emerging and common diseases, lead to revenue for pharmaceutical companies. However, without industry sponsorship of clinical trials innovation may not reach our patients [86]. Potential financial conflicts of interest (FCI) occur among all stakeholders, investigators and journal editorialists, as well as members of regulatory agencies [6, 87–91]. Studies have shown an association between the likelihood of drug endorsement in consensus guidelines and the FCI of the main author [92, 93].

## Regulatory agencies

With the advent of expedited drug approval programmes, regulatory approvals based on limited evidence (e.g. use of surrogate endpoints and non-phase 3 trials) increasingly occur when an intervention serves an ‘unmet clinical need’. This should encompass cancers that have few treatment options with poor disease outcomes [94]. Incorrect use of ‘unmet clinical need’ may, in part, explain the high rate of expedited oncology drug approvals. A recent study showed 95% of 58 FDA approvals from 2012 to 2017 entering an expedited programme [95]. In another study analysing FDA applications for new cancer drugs/indications from 2008 to 2016, 53 of 186 were granted expedited approval [96]. Recently, margetuximab, a monoclonal HER2neu-directed antibody, was granted expedited approval by the FDA for treating advanced breast cancer patients after two prior treatment lines with HER2neu-directed therapy [97]. The drug approval was dated before the publication of the clinical trial [98] and was based on a surrogate endpoint with a PFS benefit of 0.9 months. It is hard to comprehend how, for a disease with many treatment options (next to chemotherapy and hormonal therapy (if applicable) and several FDA-approved HER2neu targeting agents [55]), this would provide in an unmet clinical need warranting expedited approval.

Expedited drug approval pathways require post-approval studies to address evidence gaps. These are often delayed, and if performed, tend to have the same inappropriate characteristics as the pre-approval trials [6, 43, 44, 99–101]. A recent overview of 25 years of accelerated approvals of oncology products (all based on surrogate endpoints) showed 51 of 93 initial approvals to confirm benefit in the ensuing years. Thirty-seven (61%) of confirmation studies again used a surrogate endpoint [42]. In another report, for 93 cancer drug indications granted accelerated approval by the FDA, only 20% of confirmatory studies used OS as an endpoint; 41% studied a surrogate with the remaining confirmatory trials not yet reported [43]. Limitations in evidence can therefore persist post approval [102].

Serious side effects may become clear after widespread use after drug approval [85]. For example, real-world post-approval data of ibrutinib, used to treat chronic lymphocytic leukaemia, revealed higher than expected treatment-related cardiac deaths, which may be dose-related [103]. To date, no lower ibrutinib dose trial has been mandated. Another example is the increased risk of myelodysplastic syndrome (MDS) and acute myeloid leukaemia (AML) with the use of poly(ADP-ribose) polymerase (PARP) inhibitors detected in a recent meta-analysis of 28 RCTs and in a study of the WHO’s pharmacovigilance database [104]. This analysis was performed after several cases were detected in the longer follow-up of PARP inhibitor trials. As maintenance PARP inhibitor use in even platinum-sensitive homologous-recombination-proficient ovarian cancer patients has been approved [105], many patients will be exposed to an increased risk of MDS/AML, but with a limited evidence of PFS increase and no proven OS benefit pertaining to their ovarian cancer [106].

Discrepancies between trial inclusion criteria and regulatory agency-approved patient characteristics contribute to the exposure of patients to drugs without supporting data. For example, both enzalutamide [107] and apalutamide [108] were FDA approved for the treatment of men with non-metastatic castration-resistant prostate cancer regardless of prostate-specific antigen (PSA) doubling time, whereas the pivotal trials were limited to men with PSA doubling times of 10 months or less [109, 110]. This broadens prescribing indications to a larger group of lower-risk patients who will be exposed longer to these expensive drugs without proven benefit [111]. A recent study identified 38 approvals (2010–2018) by the FDA, EMA and Pharmaceuticals and Medical Devices Agency; 53% revealed discrepancies between trial inclusion criteria and defined therapeutic indications, and several allow broader prescribing indications [112].

## Drug pricing

Conceding the fact that drug pricing, regulatory bodies and financial thresholds for acceptable costs vary between countries, the benefit of many new cancer treatments is in stark contrast to their pricing [7, 113]. No positive association between clinical benefit scales, efficacy or novelty and pricing has been detected [30, 32, 41]. Revenue of approved drugs is such that the need for high drug prices to cover research and development costs may not always be a valid argument [114], especially since the portfolios of pharmaceutical companies overlap, limiting risk and costs of innovation [16, 115].

High drug prices not only lead to cancer treatments being limited to high-income countries but may even delay drugs reaching patients due to unavoidable time-consuming price negotiations [116]. Financial constraint is one of the main determinants of health care availability. With increasing drug prices, even in affluent countries, financial limitations influence cancer drug availability [13, 117].

## Raising the bar

Several recommendations can be made that could all contribute to raising the bar in oncology (see Box 1). The first important step is the recognition of the scope of the problems depicted above. The explosive increase in publications on new treatments renders oncologist’s dependent on rapid reading services, abstract reading and expert opinion regarding evidence assessment. A recent study showed that clinicians often overestimate the benefit and underestimate the harm of medical interventions [118]. Unrealistic patient expectations of ‘breakthrough treatments’ [11, 95] occur as a result of direct-to-consumer marketing with a poor translation of scientific data by lay press [81], cancer centres [119] and treating physicians [120, 121]. Medical education needs to be transformed so that we are better prepared for the broader responsibilities and social aspects of modern-day medicine, data interpretation, research and value-weighted treatment selection.

Educational focus on a ‘less is more’ principle for managing patients with advanced cancer should contribute to raising the bar. By adhering to QOL standards, the value of early palliative care with timely refrainment from anticancer therapy should be an integral part of oncology training. Patients suffering from advanced cancer are willing to undergo toxic treatments with a very small chance of benefit near the end of life [122]. However, systemic anticancer treatments often provide limited or no benefit to patients suffering from advanced cancer [123], lead to harm in the last weeks of life, reduce QOL, limit timely hospice care and increase risk of dying in the hospital with subsequently an increased risk of pathological grievance of bereaved [124, 125]. Informing patients of treatment effects in daily practice by quoting point estimates, such as median OS achieved in highly selected patients from RCTs instead of using ‘real-life data’ (if available), could contribute to unrealistic patient expectations [126]. Accurately disclosing prognostic information does not negatively impact patient–physician relationships but may skew low-value life-prolonging care to comfort-oriented care [127]. Therefore, translating complex trial and real-life data to daily clinical practice and adequately and understandably communicating this to patients should receive appropriate attention in oncology training.

Both the medical scientific society and regulatory agencies should aim at improving the quality of clinical trials. The use of surrogate endpoints should be limited to those for which evidence exists of their validity (e.g. metastasis-free survival in localised prostate cancer [128]). Head-to-head comparisons of new drugs and use of the standard of care in control arms should be the norm instead of comparing many new ‘same class’ drugs to inappropriate control arms. Lowering the arbitrary *p* ≤ 0.05 for rejection of the null hypothesis in RCTs has been proposed as a possible strategy to reduce the risk of false-positive results at the cost of occasionally missing a true and clinically relevant treatment effect [129, 130]. Either the *p* value for positivity should be lowered or trial replication should, whenever possible, be mandatory before regulatory approval and inclusion into guidelines.

Regulatory agencies should mandate not only confirmatory trials after rapid approvals but should also critically address post-marketing safety concerns. Expedited approval processes should be limited to indications for true unmet clinical needs. When approving drugs, both regulatory agencies and medical societies should adhere to indications as studied, thereby limiting unwarranted prescribing. Retraction of drug approvals should be followed by guideline retractions.

Bias in journal reporting should be eliminated and all data should be available at the time of issuance of both regular approvals and expedited approvals [131]. Data should be presented in a more palatable manner for both physicians and the lay press by employing measures such as absolute risk reductions, number needed to treat and number needed to harm [132]. When applicable, reporting the fragility index, as an indication of trial data robustness, should be considered [67]. Concluding statements in abstracts should be simple and clear and strictly relate to the primary endpoint. Stating FCI’s to readers and audiences should be mandatory, but FCI’s should ideally be avoided.

Our societal responsibility transcends oncological care. Implementation of costly low-value oncological treatments invariably impacts health care accessibility in other medical fields. At least in our country, for the price of one pembrolizumab dose administration [133], four type 1 diabetes mellitus patients can use a flash glucose monitoring system for almost a year to better manage their disease [134], against which they face a lifelong battle. We should, as health care advocates, scrutinise drug pricing and aim for fair distribution of health care spending. Concerted efforts are needed at all levels (physicians, clinical researchers, medical societies, drug companies, regulatory agencies, payers and politicians) for this to be effectively addressed. Integration of value-based scales into regulatory agency policies should raise the bar for drug approvals as well as influence drug pricing and availability. As oncology drugs may often be dosed higher or more frequently than needed, lower doses, alternate dosing schedules and shorter treatment times should be studied [135]. Efforts have been initiated to reduce the use of inappropriate tests, treatments, procedures and costs in oncology, such as the ‘Choosing Wisely’ initiative [136].

The COVID-19 pandemic has impacted health care resources urging the medical community to increasingly adhere to only high-value health care interventions in all medical fields. Strictly selecting high-value cancer care should carry momentum in the oncology landscape far beyond this world disruptive event.

Box 1 Recommendations to raise the bar in the field of oncology Recognition of the limitations of evidence generated in trials leading to oncology drug approvals and subsequent medical education transformation.

Increased focus in medical education on a ‘less is more’ principle with special attention for communication skills for accurate disclosure of prognostic information and aiming for the timely institution of palliative care.

Insisting on improvement in clinical trial design, conduct and execution employing valid meaningful endpoints, proper comparator arms and replication of findings or lowering of *p* values.

Journals should avoid bias in publications with clear and palatable reporting limiting abstract conclusions to the primary trial endpoint, clearly stating but preferably avoiding author/editor FCI.

Strict limitation of regulatory agency use of expedited approval procedure to true unmet clinical needs; rigorous pursuit of mandatory confirmatory high-quality trials after expedited approvals; meticulous post-marketing monitoring for safety with prompt intervention in case of concern.

Regulatory agency approval adherence to indications as studied in the pivotal clinical trial.

Concerted effort to address oncology drug pricing aiming to balance pricing with drug efficacy with fair health care distribution.

Concerted effort to study alternative, lower and less frequent dosing schedules maintaining efficacy for oncology drugs.

## Data Availability

Not applicable.
